# Vibrio cholerae lineage and pangenome diversity vary geographically across Bangladesh over 1 year

**DOI:** 10.1099/mgen.0.001437

**Published:** 2025-07-25

**Authors:** Chuhan Qin, Patrick Lypaczewski, Md. Abu Sayeed, Aline Cuénod, Lindsey Brinkley, Ashton Creasy-Marrazzo, Emilee T. Cato, Kamrul Islam, Md. Imam Ul Khabir, Md. Taufiqur R. Bhuiyan, Yasmin Begum, Manasi N. Kamat, Laura S. Bailey, Kari B. Basso, Firdausi Qadri, Ashraful I. Khan, Eric J. Nelson, B. Jesse Shapiro

**Affiliations:** 1 Department of Microbiology & Immunology, McGill University, Montréal, Canada; 2 McGill Genome Centre, McGill University, Montréal, Canada; 3 Departments of Pediatrics and Environmental and Global Health, Emerging Pathogens Institute, University of Florida, Gainesville, FL, USA; 4 Infectious Diseases Division (IDD), International Centre for Diarrhoeal Disease Research, Bangladesh (icddr, b), Dhaka, Bangladesh; 5 Alabama State University, Montgomery, AL, USA; 6 Department of Chemistry, University of Florida, Gainesville, FL, USA; 7 McGill Centre for Microbiome Research, McGill University, Montréal, Canada

**Keywords:** antimicrobial resistance, bacteriophage, cholera, long-read sequencing, pangenome, phylogenetics, *Vibrio cholerae*

## Abstract

Cholera is an acute diarrhoeal disease caused by *Vibrio cholerae*. It remains a major public health challenge worldwide, and particularly in the endemic region around the Bay of Bengal. Over decadal time scales, one lineage typically dominates and spreads in global pandemic waves. However, it remains unclear to what extent diverse lineages co-circulate during a single outbreak. Defining the pool of diversity over finer time-scales is important because the selective pressures that impact *V. cholerae*, namely antibiotics and phages, are dynamic on these scales. To study the nationwide diversity of *V. cholerae*, we long-read sequenced 273 *V*. *cholerae* genomes from seven hospitals over 1 year (2018) in Bangladesh. Four major *V. cholerae* lineages were identified: three known lineages, BD-1, BD-2a and BD-2b, and a novel lineage that we call BD-3. In 2022, BD-1 caused a large cholera outbreak in Dhaka, at which point it had replaced BD-2 as the most common lineage in Bangladesh. We show that, in 2018, BD-1 was already predominant in the five northern regions, including Dhaka, consistent with an origin from northern India. By contrast, we observed a higher diversity of lineages in the two southern regions near the coast. The four lineages differed in pangenome content, including integrative and conjugative elements (ICEs) and genes involved in resistance to bacteriophages and antibiotics. Notably, BD-2a lacked an ICE and is predicted to be more sensitive to phages and antibiotics, yet persisted throughout the sampling period. Genes previously associated with antibiotic resistance in *V. cholerae* isolated from Bangladesh in the prior decade were entirely absent from all lineages in 2018–2019, suggesting shifting costs and benefits of encoding these genes. Our results highlight the diverse nature of the *V. cholerae* pangenome and geographic structure within a single outbreak season. This diversity provides the raw material for adaptation to antibiotics, phages and other selective pressures.

**Impact Statement**

Since the start of the seventh cholera pandemic in the 1960s, successive waves of outbreaks have been associated with successive predominant lineages of *Vibrio cholerae*. However, the extent and dynamics of lineage diversity over finer spatial and temporal scales remain unclear. To fill this gap, we sequenced 273 *V. cholerae* genomes sampled from seven locations across the cholera-endemic region of Bangladesh over 1 year. We identified four major lineages of *V. cholerae* that differed in their gene content, including antimicrobial and phage resistance genes. We found greater variation in genomic diversity over space than time, with higher diversity observed in more southern locations. This observation is consistent with a dominant *V. cholerae* lineage having been introduced from the northern border with India. On a technical level, we demonstrate the potential of long-read nanopore sequencing for genomic epidemiology studies. Our results broadly support a model in which local selective pressures, including phages and antibiotics, maintain pathogen diversity within a region and outbreak season, even if this diversity is lost through successive lineage replacements over decadal scales.

## Data Summary

All DNA sequences generated in this project are available in the National Center for Biotechnology Information GenBank database under BioProject PRJNA1174068. Individual accession numbers are listed in Table S1.

## Introduction

Cholera is an acute bacterial infectious disease characterized by profuse watery diarrhoea. It is caused by the Gram-negative facultative pathogen *Vibrio cholerae*, which can be found in coastal brackish waters in planktonic forms or attached to zooplankton or shellfish [1,2]. *V. cholerae* is genetically diverse in the aquatic environment, and only two serogroups (O1 and O139) are associated with pandemic cholera [2]. The current seventh pandemic was first detected in 1961 in Indonesia, caused by a *V. cholerae* lineage referred to as the seventh Pandemic El Tor (7PET), which is genetically distinct from the classical type responsible for the sixth pandemic [3]. The public health burden of cholera remains high, with 2.9 million cases and 95,000 deaths estimated to be caused by cholera every year [4]. The burden of cholera is likely underestimated because cases are often unreported for geo-political and logistical reasons; infections can also be asymptomatic and less likely to be detected, especially in endemic settings [5]. A recent study based on serological surveillance showed that the *V. cholerae* infection rate may be much higher – at 535 per 1,000 people annually in Bangladesh [5]. Outside the endemic region around Bangladesh, shorter but often devastating outbreaks can occur, as demonstrated in Haiti in 2010 or in Yemen in 2016 [3,6]. Over global geographic scales and decadal time scales, *V. cholerae* evolution is dominated by successive lineages outcompeting others in consecutive pandemic waves [3]. Despite some studies on regional or even municipal scales [7], *V. cholerae* evolution on finer temporal and geographic scales is less well understood. This knowledge gap is important when evaluating resiliency at the population level to varied antimicrobial exposures that may shift within these short time scales.

Two major sub-lineages of 7PET, known as BD-1 and BD-2, have co-circulated in South Asia since the late 1990s, where ‘BD’ stands for Bangladesh [8]. Despite the naming, up until 2017, the Indian population contained mostly BD-1, whereas BD-2 predominated in Bangladesh [8,9]. In 2022, Bangladesh experienced one of the largest cholera outbreaks in recent years when BD-2, which had been dominant in Bangladesh since the early 2010s, was superseded by BD-1.2 [8,10]. The BD-1.2 lineage is most closely related to BD-1 genomes from India, suggesting a cross-border origin in India [8]. BD-1 and BD-2 are relatively closely related lineages within the 7PET. They differ by single-nucleotide changes and gene content differences in pathogenicity islands such as VSP-II and integrative and conjugative elements (ICEs). It remains unclear which, if any, of these differences explain why one lineage might replace another and to what extent lineages differ in their relative abundances across Bangladesh.

VSP-I and VSP-II are two pathogenicity islands specific to 7PET genomes [9]. The structure of VSP-I is stable across 7PET from recent outbreaks, whereas five different types of VSP-II were identified in BD-1 and BD-2. Recent studies have associated genes on VSP-II with functions such as chemotactic responses, cell congregation and phage defence [11,12].

ICEs encode numerous antimicrobial resistance (AMR) genes and were recently shown to confer resistance to the lytic ICP1 bacteriophage through diverse phage defence genes [13,14]. The first ICE discovered in *V. cholerae* was named SXT, as it conferred resistance to sulfamethoxazole and trimethoprim [15,16]. ICE variants encode different AMR and phage resistance genes. For example, among *V. cholerae* genomes from 2006, the genes *qnr_Vc_* (encoding a pentapeptide that protects DNA gyrase from the attack of ciprofloxacin) and *mphA* (encoding a phosphotransferase that inactivates azithromycin) were, respectively, associated with ciprofloxacin and azithromycin resistance phenotypes localized on an ICE [17]. Frequent homologous recombination between ICEs is thought to occur, allowing rapid gain and loss of AMR genes [18]. The same is likely true of phage-resistance genes. Notably, the *Vch*Ind5 ICE variant, but not the *Vc*Ind6 ICE, encodes a BREX anti-phage system that is associated with lower phage to *V. cholerae* ratios within patients [13,14]. Some *V. cholerae* genomes lack an ICE entirely, suggesting fitness costs under certain conditions.

To determine the distribution of *V. cholerae* lineages and genes circulating in Bangladesh, we sequenced 273 *V*. *cholerae* genomes collected in 2018 and early 2019 using a ‘long-read’ method from seven hospitals across the country, spanning a single annual outbreak season. In addition to the previously described BD-1 and BD-2 lineages, we identified two BD-2 sub-lineages, one of which typically lacks an ICE, and a novel lineage, BD-3, which was restricted to Dhaka in our data set. Despite the higher prevalence of BD-1, BD-2 persisted at low frequency during the sampled period. BD-1 was more prevalent in northern Bangladesh bordering India, supporting its Indian origin. Southern regions contained a higher diversity of lineages, potentially due to distinct environments and migration patterns. We also highlight the diversity of AMR and phage-resistance genes, many of which occur on the ICE and differ across lineages. Two genes associated with antibiotic resistance in 2006 were absent from all *V. cholerae* genomes in our 2018–2019 sample, exemplifying the dynamic nature of the pangenome.

## Methods

### Ethics statement

Stool samples for this study were collected as part of two published IRB-approved clinical studies in Bangladesh: (i) the mHealth Diarrhoea Management (mHDM) cluster randomized controlled trial (IEDCR IRB/2017/10; icddr,b ERC/RRC PR-17036; University of Florida IRB 201601762) [19] and (ii) the National Cholera Surveillance (NCS) study (icddr,b ERC/RRC PR-15127) [20].

### Study design

A prospective longitudinal study of patients presenting with diarrhoeal disease was conducted at five Bangladesh Ministry of Health and Family Welfare district hospitals (both mHDM and NCS sites) and two centralized exclusive NCS hospitals (BITID; icddr,b). Sites were distributed geographically nationwide [13]. For mHDM, the inclusion criteria were patients with acute uncomplicated diarrhoea (less than 7 days with at least three loose stools in the last 24 h) and age greater than or equal to 2 months; four patients per study day were sampled. For NCS, the case definitions differed by age. For age under 2 months: changed stool habit from the usual pattern in terms of frequency (more than the usual number of purging) or nature of stool (more water than faecal matter). For age 2 months and over: three or more loose or liquid stools within 24 h or three loose/liquid stools or fewer causing dehydration in the last 24 h. Enrolment per study day was two patients with diarrhoea aged less than 5 years old and two patients aged 5 years or older; if the target number of patients in a particular age group was not met, the study overenrolled in the other group to meet the target of four patients per day during workdays. There were no exclusions (mHDM or NCS) based on prior reported antibiotic exposure.

### Bacterial culture

Stool samples were collected at hospital admission. Aliquots for transport and subsequent culture were stabbed into Cary–Blair transport media. The culture was performed via standard methods with both thiosulfate–citrate–bile salts–sucrose agar and taurocholate–tellurite gelatine agar [20]. Suspected *V. cholerae* colonies were serotyped with antibodies specific to O1 (Inaba and Ogawa) and O139 serogroups [20]. Isolates were stored in glycerol at −80 °C at the icddr,b, shipped on dry ice to the University of Florida and re-isolated on Luria–Bertani (LB) agarose plates for nucleic acid analysis and storage at −80 °C in glycerol.

### DNA extraction and sequencing

DNA extraction was performed on one pure colony grown in 5 ml of LB broth at 37 °C at 220 r.p.m. DNA was extracted using the DNeasy Blood and Tissues kit following the manufacturer’s instructions (QIAGEN), followed by quantification using a NanoDrop spectrophotometer (Thermo Fisher Scientific) and Qubit fluorometer (Thermo Fisher Scientific). For each sample, ~200 ng was used to generate the sequencing library using the Nanopore Rapid Barcoding Kit 96 V14 (SQK-RBK114.96, Oxford Nanopore Technologies) following the manufacturer’s instructions. Briefly, the samples were adjusted to 10 µl with DNase-free water, and 1 µl of rapid barcodes was added to each sample in groups of 96. The plates were incubated for 2 min at 30 °C, followed by enzyme inactivation for 2 min at 80 °C. Two microlitres per sample were used to pool the plates, followed by concentrating using a 1:1 AMPure XP Beads clean up (Beckman Coulter). Beads were rinsed using two washes of 80% ethanol with 180° turns. The samples were eluted in 12 µl elution buffer EB (Oxford Nanopore Technologies). Eleven microlitres of the pooled library were ligated to 1 µl of pre-diluted Rapid Adapter and loaded on a PromethION R10.4.1 flow cell following priming (Oxford Nanopore Technologies). Whole-genome sequencing was performed on a PromethION P2 Solo long-read sequencer with live base-calling enabled and Fast mode to monitor sequencing progress (Oxford Nanopore Technologies). The resulting Fast5 files were merged and converted to the POD5 format using Pod5 v.0.2.4 (Oxford Nanopore Technologies). The POD5 files were base-called and demultiplexed per plate using guppy v.6.4.6 (Oxford Nanopore Technologies) using the *dna_r10.4.1_e8.2_400bps_sup* model with barcode and adapter trimming and a minimum *Q*-score of 7. Four samples from the 277 collected failed to produce sufficient sequencing data and were removed from all downstream analyses.

### Phylogenetic analysis

Reads passing filtering for each demultiplexed sample were assembled using Flye v.2.9.1 using the option *-nano-hq* [21]. The contigs were scaffolded for consistency using RagTag v.2.1.0 [22]. To obtain a phylogeny using high-confidence single-nucleotide variants (SNVs), reads were mapped to reference genome N16961 (RefSeq ID: GCF_900205735.1) using medaka v.1.11.3 (which employs minimap2 for mapping) [23,25]. Indels and SNVs with QUAL scores lower than 40 were removed, where the QUAL score stands for the Phred-transformed error probability predicted by the recurrent neural network of medaka. Short sequence reads of 776 publicly available 7PET genomes from around the world were additionally aligned to N16961 using BWA-MEM, and variants were called using GATK HaplotypeCaller [26,27]. Homologous recombination was inferred using Gubbins v.3.2.1, allowing us to estimate the ratio of recombination (r) to mutation (m) among core genome SNVs [28].

The SNVs called by medaka were used to construct a maximum likelihood phylogenetic tree using RAxML v.8.2.12 with a bootstrap analysis over 500 replicates and with the GTRCAT model of nucleotide substitution [29]. A 7PET isolate from 1971 Bangladesh (ERR025385) was used as the outgroup to root the tree [3]. The assemblies were clustered using PopPUNK v.2.4.0, which compares the similarity of the whole genome *k-mer* set [30]. The core genomes of these assemblies were also clustered using fastBAPS v.1.0.8 [31].

The phylogenetic tree, combined with a heatmap showing the presence of ICEs and the geographical distribution, was visualized in R using the package ggtree [32]. PastML was used to infer the most likely ancestral states (country of origin) for internal branches [33]. Bayesian phylogenetic inference was performed on the BD-1.2 sub-lineage using BEAST v.10.5.0, assuming a constant population size coalescence model with a strict clock rate, a generalized time-reversible substitution model and a *γ* distribution of base substitution heterogeneity [34]. The length of the Markov chain Monte Carlo chain was set at 10,000,000.

### Identification of integrative conjugative elements

The assemblies were screened for the presence of a set of known SXT-ICEs using blastn [14,35]. The accession numbers for different ICE types are available in Supplementary TableData S5 of Legault *et al*. [14]. An ICE was called as present if at least 90 % of its sequence was covered in an isolate and the average identity of aligned sequences was larger than 95%.

### Serotype inference

Ogawa or Inaba serotypes were inferred by comparing the *wbeT* sequences. Ogawa isolates contain an intact copy of the *wbeT* gene, whereas Inaba isolates contain a dysfunctional *wbeT* [36]. Therefore, the Inaba serotype was predicted if the *wbeT* sequence of an isolate was disrupted by insertions or contained mutations known in the literature to render it dysfunctional [36,38]. Inserted sequences were annotated by querying the GenBank non-redundant protein sequences database using blastx [35].

### Pangenome analyses

Panaroo v.1.1.2 was used to estimate the pangenome of each *V. cholerae* lineage of interest [39]. To measure the openness of pangenomes in BD-1 compared to BD-2, the model from Tettelin *et al*. using Heap’s law was fitted: $n\sim N^{\gamma }$, where n is the number of total genes and N is the number of genomes sampled [40]. A higher *γ* value implies a higher rate of increase in gene numbers with an increase in genome numbers, hence a higher average divergence in genetic content. Nei’s distance (*π*) was used to measure pairwise core distances, whereas Jaccard’s distance of the gene contents was used to measure the accessory distances. This was followed by plotting the distribution of accessory distances in BD-1 and BD-2 in a boxplot binned by core distances.

### Screening for known AMR genes

Known AMR genes in the Comprehensive Antibiotic Resistance Database (CARD) were screened in the *de novo* genome assemblies using CARD-RGI [41]. To investigate deletions of AMR genes on ICEs, the ICE sequences were obtained by extracting the intervals between the two flanking genes, *prfC* (N16961_RS11085) and *gmr* (N16961_RS11090). Pairwise comparisons of ICE sequences were generated using blast and visualized with the Artemis Comparison Tool [35,42].

### Screening for known phage-defence mechanisms

The presence of known phage-defence mechanisms in the *de novo* genome assemblies was inferred using DefenseFinder v.1.3.0 [43].

### Detection of antibiotics in stool by liquid chromatography–mass spectrometry

Ciprofloxacin, doxycycline, and azithromycin were detected and quantified from stool samples by liquid chromatography–mass spectrometry as previously described [13].

### Statistical analyses and visualisation

Statistical analyses and visualisation were done using R. The R code used for plotting the figures is available from https://github.com/Chuhan-Qin/Vibrio_2024.

## Results

### Sample collection

A total of 277 *V*. *cholerae* isolates were obtained from stool samples collected as part of the nationwide study [16]. One isolate was taken from each patient. We previously reported shotgun metagenomic sequencing from the same set of patients who varied in dehydration severity from mild to severe [13].

### Long-read sequencing yields mostly complete genome assemblies

We successfully sequenced genomes from 273 of the 277 isolates using Nanopore technology (‘Methods’). The median read N50 across samples was 6,598 bp, with a maximum of 43,509 bp. The reads were assembled into genomes ranging from 4.0 to 4.1 Mbp, consistent with the known range of *V. cholerae* genome sizes. The assembly sizes clustered around either 4.0 or 4.1 Mbp with very few in between (Fig. S1). Most genomes (180/273; 65%) were assembled into two circular contigs, corresponding to the two complete chromosomes of *V. cholerae*. In a recent study, we detected prevalent chromosome fusion in BD-2 genomes long-read sequenced from Dhaka between 2015 and 2018 [44]. In the 2018–2019 genomes reported here, we found no clear instances of fusion based on the assemblies. Based on mapping raw reads, we found two isolates (EN_1554 and EN_1688) each containing a single read spanning one of the two fusion sites. Both these isolates assembled into two circular chromosomes, indicating that fusion was unlikely or present in a small subpopulation only. The 273 genomes were assembled into seven contigs or fewer per genome, a marked improvement over the average of 73 contigs per genome in a recent study using short-read Illumina sequencing [45]. Similarly, *V. cholerae* assemblies in the ENA database contained a median of 92 contigs, and only 202 of the 9950 genomes (2.06%) were assembled into two circular contigs (accessed on 30 August 2024). In addition to the two circular chromosomes, we found only one other circular contig of 120 Kbp in isolate EN-1738, which was identified as an ICP1 phage.

### Major *V. cholerae* lineages identified in the phylogeny

**Fig. 1.**
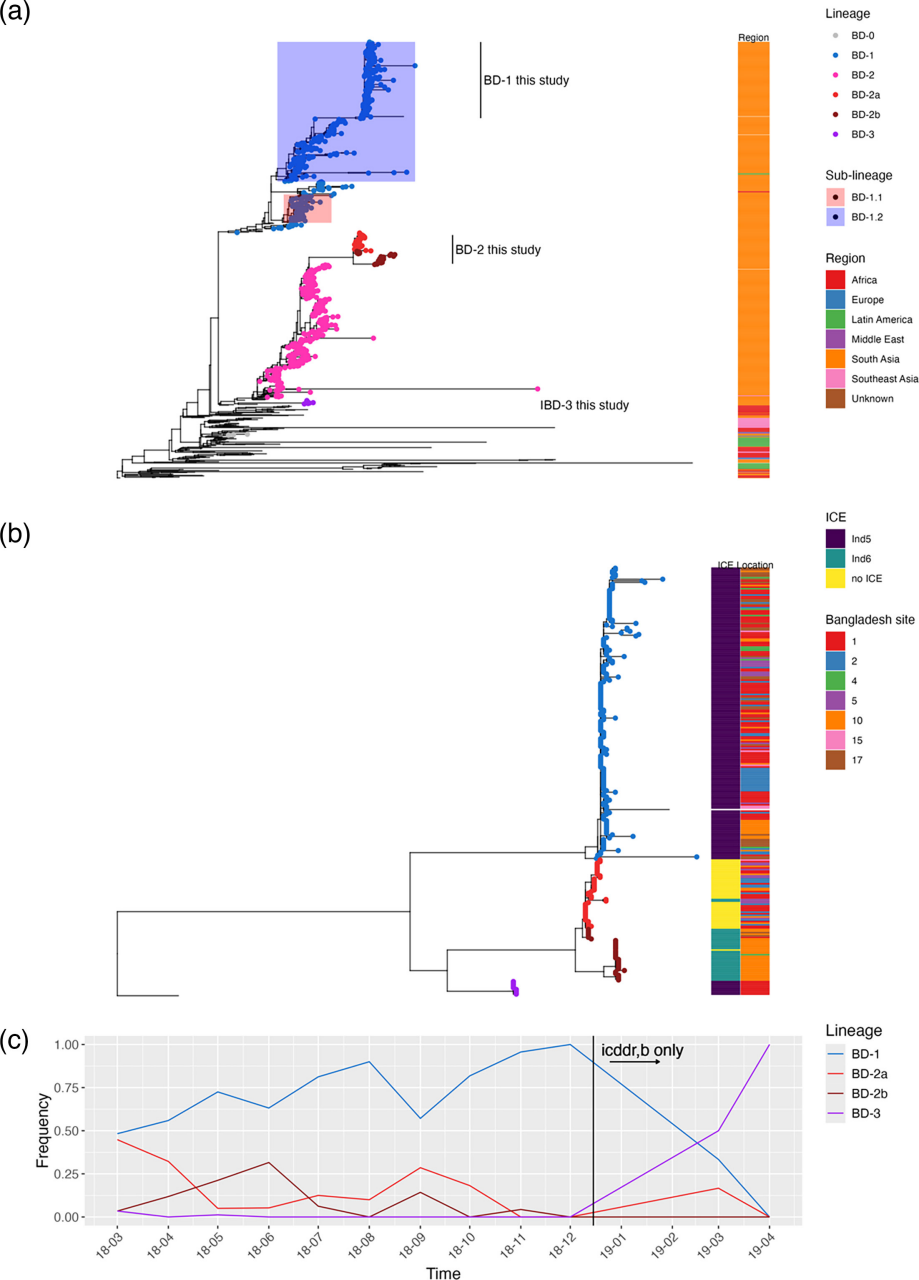
Major phylogenetic lineages of *V. cholerae* sampled across Bangladesh. (**a**) Phylogenetic tree of newly sequenced *V. cholerae* genomes in the context of 776 publicly available genomes. The maximum likelihood tree was rooted with a 1971 Bangladesh 7PET isolate as the outgroup (ERR025385). Leaves are coloured according to phylogenetic lineages: blue for BD-1, different shades of red for sub-lineages of BD-2 and purple for BD-3. Sub-lineages of BD-1 corresponding to BD-1.1 and BD-1.2, as defined in previous studies, were shaded. The heatmap shows the geographical regions and the year of sampling. (**b**) Association between major lineages, ICE types, and sampling sites within Bangladesh. The same outgroup ERR025385 and leaf colouring scheme were used as in panel (**a**). Other than the outgroup, only the 273 genomes sequenced in this study are included. The heatmap shows the presence of SXT-ICEs and the locations from which isolates were sampled within Bangladesh. (**c**) Frequency of phylogenetic lineages over time. The *X*-axis shows the time of sampling. The *Y*-axis shows the relative proportion of lineages at each time point. Note that the isolates in 2019 were from icddr,b only.

To place our genomes in the context of known *V. cholerae* diversity, 776 publicly available *V. cholerae* 7PET genomes were downloaded from ENA and aligned with our genomes to construct a maximum-likelihood phylogeny (Table S1). The tree was rooted with ERR025385, a 7PET isolate sampled from Bangladesh in 1971 [3]. We found a median of 22 pairwise SNV differences among the 273 newly sequenced genomes, consistent with relatively little genetic diversity within 7PET. Most of these genomes fall within two lineages that have been co-circulating in Bangladesh since the 1990s, BD-1 and BD-2 (Fig. 1a) [9]. Consistent with little detectable homologous recombination in 7PET [3], the inferred r/m in our data set is below 0.01, unlikely to have any major effects on the phylogeny.

We identified nine genomes forming a distinct monophyletic group (henceforth referred to as BD-3), which branches near the root of BD-2 (Fig. 1a). The publicly available genomes genetically closest to BD-3 are BD-2 isolates from the early 2000s, suggesting that BD-2 and BD-3 shared a common ancestor around that time. These lineages, based on SNVs in the core genome, showed a high concordance with the whole-genome *k-mer* clustering using PopPUNK (‘Methods’). PopPUNK genomic cluster 1 (GC-1) corresponded to BD-1 (coloured blue in Fig. 1), whereas BD-2 was split into GC-2 and GC-3 (coloured shades of red in Fig. 1, henceforth referred to as BD-2a, BD-2b).

To support the phylogenetic relationships among *V. cholerae* lineages at a higher resolution, a second maximum likelihood tree was constructed using the 273 newly sequenced genomes only (Fig. 1b). Both trees identified BD-1, BD-2 and BD-3 as distinct monophyletic groups with bootstrap support of 100 %. Consistent with our earlier metagenomic sequencing, two types of ICEs, *Vch*Ind5 and *Vch*Ind6, are found in the isolate genomes [13]. All except two BD-2a isolates lacked an SXT-ICE, consistent with the shorter median length of BD-2a assemblies, which was 91,825 bp shorter than that of BD-2b (Welch two-sample *t*-test: *P*<2.2×10^−16^), close to the average size *Vch*Ind6 at 94,599 bps (Fig. S1). Other than occasional ICE gain or loss events, the ICE type is generally a stable feature within each lineage (Fig. 1b), with BD-1 and BD-3 containing *Vch*Ind5 and most BD-2b genomes containing *Vch*Ind6.

BD-1 and BD-2 also varied in their inferred immunological properties. Inaba and Ogawa are the two serotypes within *V. cholerae* O1 El Tor. They differ in the structure of the O-antigen in their lipopolysaccharides: Ogawa serotypes primarily express A and B antigens with a small amount of C antigens, whereas Inaba serotypes only express A and C antigens [36,46]. The differences in serotype are not yet known to affect virulence or virulent phage sensitivity but are clear epitopes for the immune response to *V. cholerae* [46]. Serotype switching from Ogawa to Inaba can occur when the WT *wbeT* is disrupted by insertions or mutations (‘Methods’). No specific point mutations known to change serotypes were identified in the genome reported here. However, we identified an insertion sequence, ISSpu7, in the *wbeT* gene of all newly sequenced BD-2 isolates. As a result, all BD-2 isolates were predicted to have an Inaba serotype, whereas all but one BD-1 isolate had an intact *wbeT* copy and predicted Ogawa serotype. According to another report, BD-1 later switched again to Inaba in 2020 [10]. The computationally inferred serotypes are largely concordant with antibody assays (‘Methods’): of the 185 BD-1 isolates containing an intact *wbeT* gene sequence, 173 (93.5 %) were experimentally typed Ogawa, 1 BD-1 isolate had disrupted *wbeT* and was experimentally typed Inaba, while the remaining 11 isolates had intact *wbeT* and were typed Inaba. Of the 78 BD-2 isolates, 71 (91.0 %) were experimentally typed Inaba (able S2).

BD-1.2 was previously associated with a massive cholera outbreak in 2022 and is thought to be more virulent [10]. However, systematic comparisons of disease severity across BD sub-lineages are lacking. We found that dehydration status is strongly associated with lineage identity in our data set (*χ*^2^ test: *P*=0.033). BD-1 is associated with more severe dehydration (standardized residuals >2) and BD-2a with mild dehydration (standardized residuals < −2). With the caveat of low sample size and its observation only in Dhaka, BD-3 is tentatively associated with more severe dehydration (Fig. S2).

### Geographical and temporal distribution of *V. cholerae* genomic diversity

**Fig. 2.**
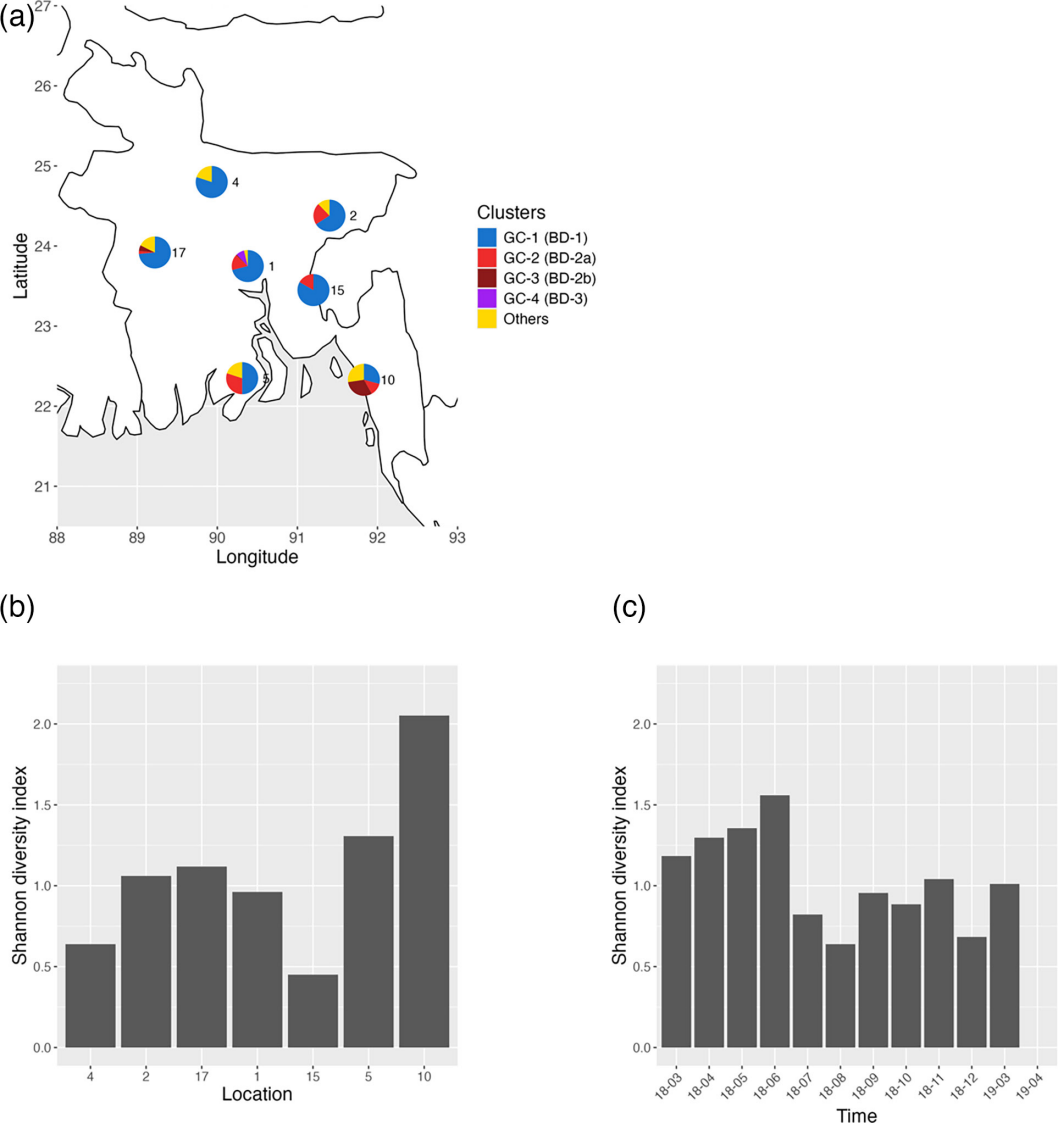
(**a**) Geographical distribution of *V. cholerae* genomic clusters across Bangladesh. Isolates are coloured by PopPUNK genomic clusters, matching with lineages coloured as in Fig. 1. ‘Others’ denotes rare clusters only observed once. (**b**) Shannon diversity of genomic clusters by location. Locations are sorted by latitude from the north to the south (left to right). (**c**) Shannon diversity of genomic clusters by month. The *X*-axis shows the sampling period in chronological order from March 2018 (18-03) to April 2019 (19-04). Note that the final time point was only sampled from Dhaka and only contained BD-3, resulting in a Shannon diversity value of 0.

To quantify the genomic diversity of *V. cholerae* between time points and locations, we compared the Shannon diversity of the PopPUNK genomic cluster (GC) composition. The GCs largely map to phylogenetic lineages: GC-1 to BD-1, GC-2 to BD-2a, GC-3 to BD-2b and GC-4 to BD-3 (Fig. S3). However, in addition to the core genome SNVs used to construct the phylogenetic trees, PopPUNK also uses accessory genome content, resulting in higher genetic resolution: 39 GCs compared to only 4 major phylogenetic lineages. For example, a genome with distinct gene content would be classified as a separate GC, despite branching within BD-1 or BD-2 (Fig. S3). Such gene content ‘outliers’ tend to be rare. They were considered together as ‘others’ for visualization purposes but counted separately to calculate Shannon diversity (Fig. 2).

The distribution of *V. cholerae* GCs varied among sampling locations across Bangladesh (Fig. 2a, b). The sites in the north, close to the Bangladesh-India border, contained predominantly BD-1.2 (GC-1, >70 %). By contrast, we observed a higher diversity of lineages in the two southern sites on the coast of the Bay of Bengal, marked by a mixture of BD-1 (GC-1) and BD-2 (GC-2 and GC-3). Site 10, which is located near a refugee camp at Cox’s Bazar, was also distinct in its lineage composition, containing a significant number of both BD-2a (GC-2) and BD-2b (GC-3) and high Shannon diversity (Fig. 2b), whereas most other sites contained only one or the other [47]. BD-3 (GC-4) was found only in Dhaka.

Previous work inferred an Indian origin for BD-1.2 in Bangladesh [9], which is consistent with the enrichment of BD-1.2 in parts of Bangladesh physically closer to India (Fig. 2a). To more formally infer the divergence time of BD-1.2, we used Bayesian ancestral reconstruction to infer the date of its most recent common ancestor and estimated the maximum likelihood of its country of origin (‘Methods’). Further supporting an Indian origin, all BD-1 isolates in this study clustered with publicly available BD-1.2 genomes and were genetically closer to Indian BD-1 isolates from the early 2010s than to Bangladeshi isolates of the same time (Fig. S4). The most recent common ancestor of the branch containing Bangladesh BD-1.2 isolates was descended from a branch of inferred Indian origin (maximum likelihood marginal estimation: *P*=99.67 %). We estimated the most recent common ancestor of BD-1.2 in 2002 (95 % highest posterior density interval: 2000–2003). While these results are consistent with an Indian origin of BD-1.2 in Bangladesh, they do not exclude possible introduction via an unsampled intermediate country, or circulation between countries rather than a simple unidirectional flow.

*V. cholerae* genomic diversity varied less over time (sd=0.41, Fig. 2c) than over sampling locations (sd=0.52, Fig. 2b). Samples from 2019 all came from Dhaka, and those samples from April 2019 had a Shannon diversity of zero as all isolates from that month were BD-3. When excluding the 2019 samples, the standard deviation of Shannon diversity over time is only 0.30, much less than the variation over space. A shift from BD-2 to BD-1 was previously reported in Dhaka from 2018 to 2019 [10]. We also observe a higher frequency of BD-1, particularly at later time points during our sampling period (Fig. 1c), suggesting that the shift towards BD-1 may be a nationwide phenomenon. When examining the wider collection of publicly available genomes (Table S1), we found that shifts in the dominant lineage had occurred previously in Bangladesh – from predominantly BD-1 in 2010 (90.9 %) to a mixture of BD-1 and BD-2 in 2011–2012, followed by a predominantly BD-2 population (96.7%) between 2013 and 2017. Therefore, the alternating dominance of BD-1 and BD-2 could be explained by fluctuating or frequency-dependent selection rather than a directional lineage replacement.

### Lineage BD-2 has a more open pangenome than BD-1

**Fig. 3.**
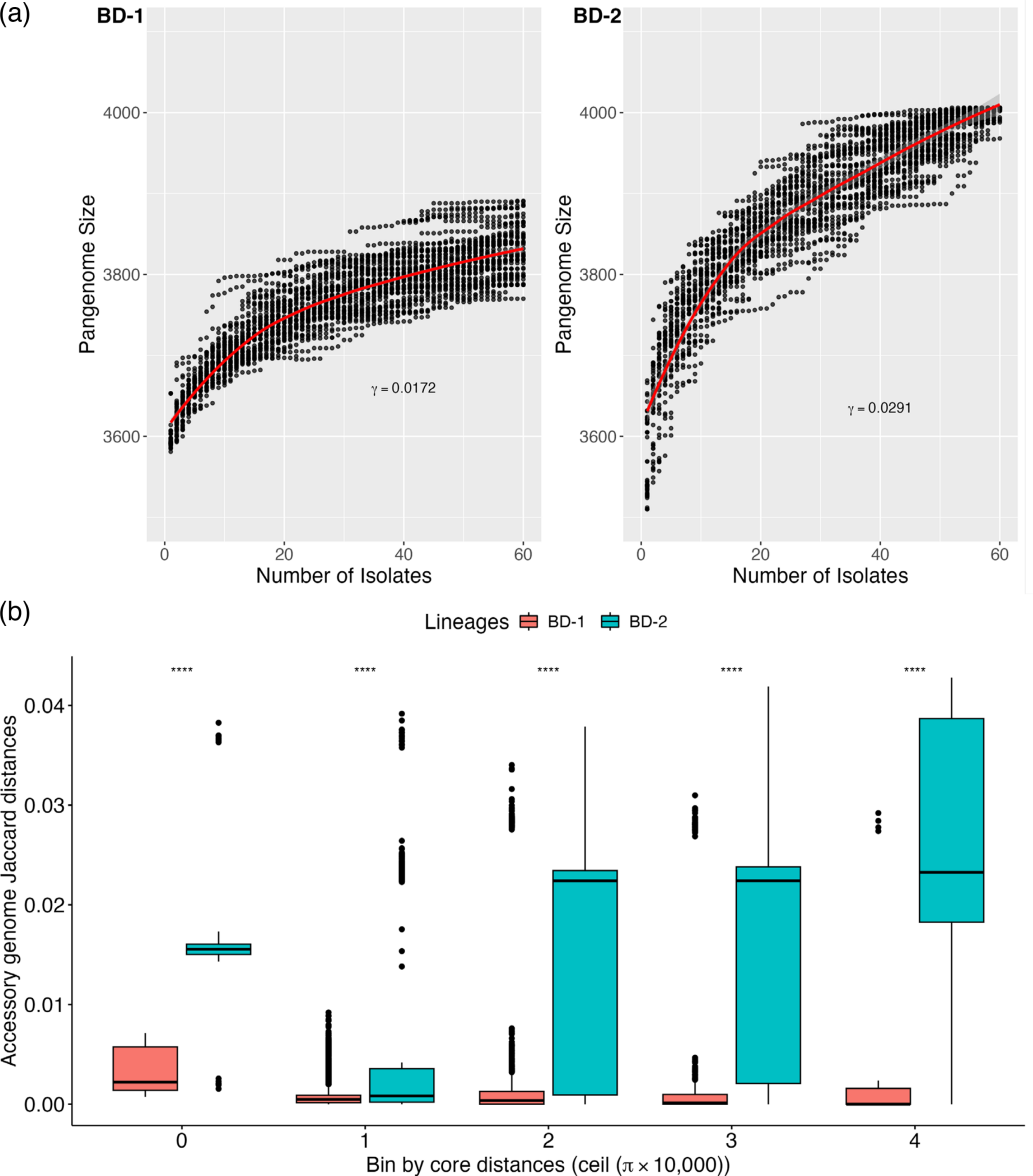
BD-2 encodes a more diverse ‘open’ pangenome than BD-1. (**a**) Pangenome size grows faster with sampling effort in BD-2 than in BD-1. The *X*-axis shows the number of isolates in each sub-sample. The *Y*-axis shows its corresponding pangenome size (number of genes). For each lineage, 50 subsets were sampled (black points). The red line shows the median. γ is the index in Tettelin’s model: $n\sim N^{\gamma }$; a higher value indicates a more open pangenome. (**b**) Distribution of within-lineage accessory distances in different core genome distance bins. The bins were set according to the core genome Nei’s distances (*π*). The box plots show the range of the within-lineage accessory genome Jaccard distances. Boxes were coloured in either red (BD-1) or green (BD-2). ‘****’ denotes a *P*<2.2×10^−16^ using the t-test.

Next, we compared the accessory gene content of BD-1 and BD-2. A pangenome is defined here as the entire set of genes from all isolates within a lineage. We found that BD-2 had a more ‘open’ pangenome (Tettelin’s γ = 0.0291), with more genes added to the pangenome per sequenced genome compared to BD-1 (γ = 0.0172, Fig. 3a). Pairwise Jaccard distances between accessory gene content were consistently higher in BD-2, and this was true across all core genome distance bins (Welch two sample t-test: *P*<2.2×10^−16^, Fig. 3b). This implies that the differences in pangenome variation are not due to differences in phylogenetic depth between BD-1 and BD-2. The high level of pangenome variation within BD-2 is likely due to its subdivision into BD-2a, which lacks an ICE, and BD-2b, which contains the *Vch*Ind6 ICE.

### Variation in AMR gene profiles

**Fig. 4.**
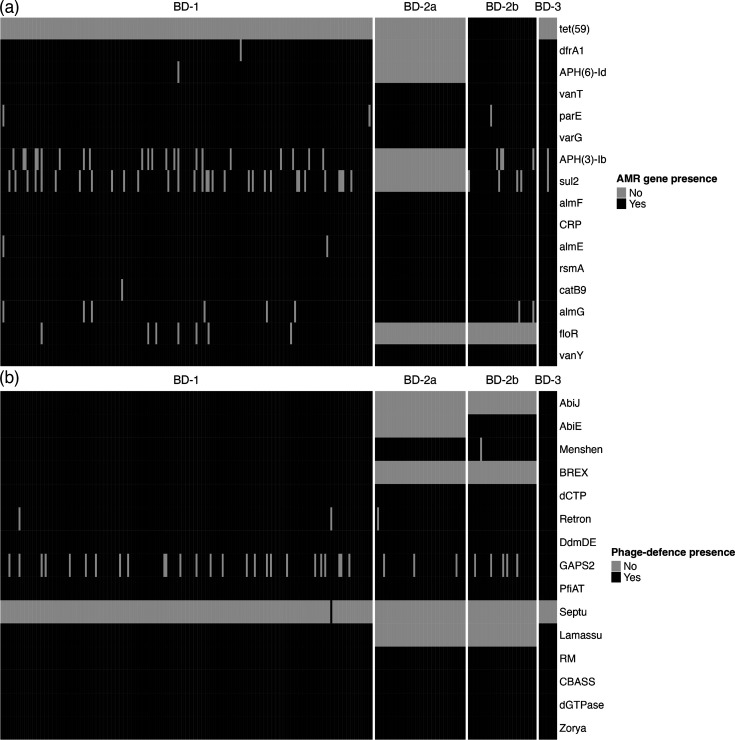
Distribution of AMR and phage-defence genes varies across major lineages of *V. cholerae*. The *X*-axis shows the isolates and is sorted by lineages. (**a**) The *Y*-axis shows the 16 AMR genes from the CARD that occurred at least once in the genome assemblies [34]. (**b**) The *Y-*axis shows the 15 phage-defence genes identified by DefenseFinder in the genome assemblies [36]. For both panels, black represents the presence, whereas grey indicates the absence.

We used the CARD to identify annotated AMR genes in our sample of genomes. We found 16 known AMR genes present in 1 or more genomes using the strict matching criteria (Table S3). The major lineages had distinct presence/absence profiles of these 16 genes (Fig. 4a). Multidrug efflux pump *rsmA* (for fluoroquinolone, diaminopyrimidine and chloramphenicol) was found in all 273 genomes, as was CRP, a global regulator for the efflux pump MdtEF, which removes fluoroquinolone, macrolide and beta-lactam from bacterial cells [48,49]. We note that the screening for CRP did not consider a point mutation that renders it dysfunctional [49]. The loss of the SXT-ICE element in BD-2a profoundly affected its AMR gene profile: almost all genomes other than BD-2a contain APH [6]-Id or APH [3]-Ib (>99 %), a phosphotransferase of aminoglycoside, which is completely absent in BD-2a (Fig. 4a). The same pattern was observed for the sulphonamide and trimethoprim resistance genes, *dfrA* and *sul2*, both of which were absent in BD-2a but had high frequencies in the other lineages. AMR genes against vancomycin (*vanY* and *vanT*), carbapenem (*varG*), fluoroquinolone (*parE*) and colistin (*almEFG*) were also prevalent across lineages (>95 %). A chloramphenicol acetyltransferase involved in chloramphenicol inactivation, *catB9*, was present in all but one BD-1 isolate. The chloramphenicol efflux pump *floR* is a hallmark of *Vch*Ind5; it was absent in BD-2 but present in almost all BD-1 and BD-3 isolates. Consistent with previous findings, *tetA*, an efflux pump for tetracycline, is located on the *Vch*Ind6 ICE and is present in all BD-2b but none of the other genomes [17].

**Fig. 5.**
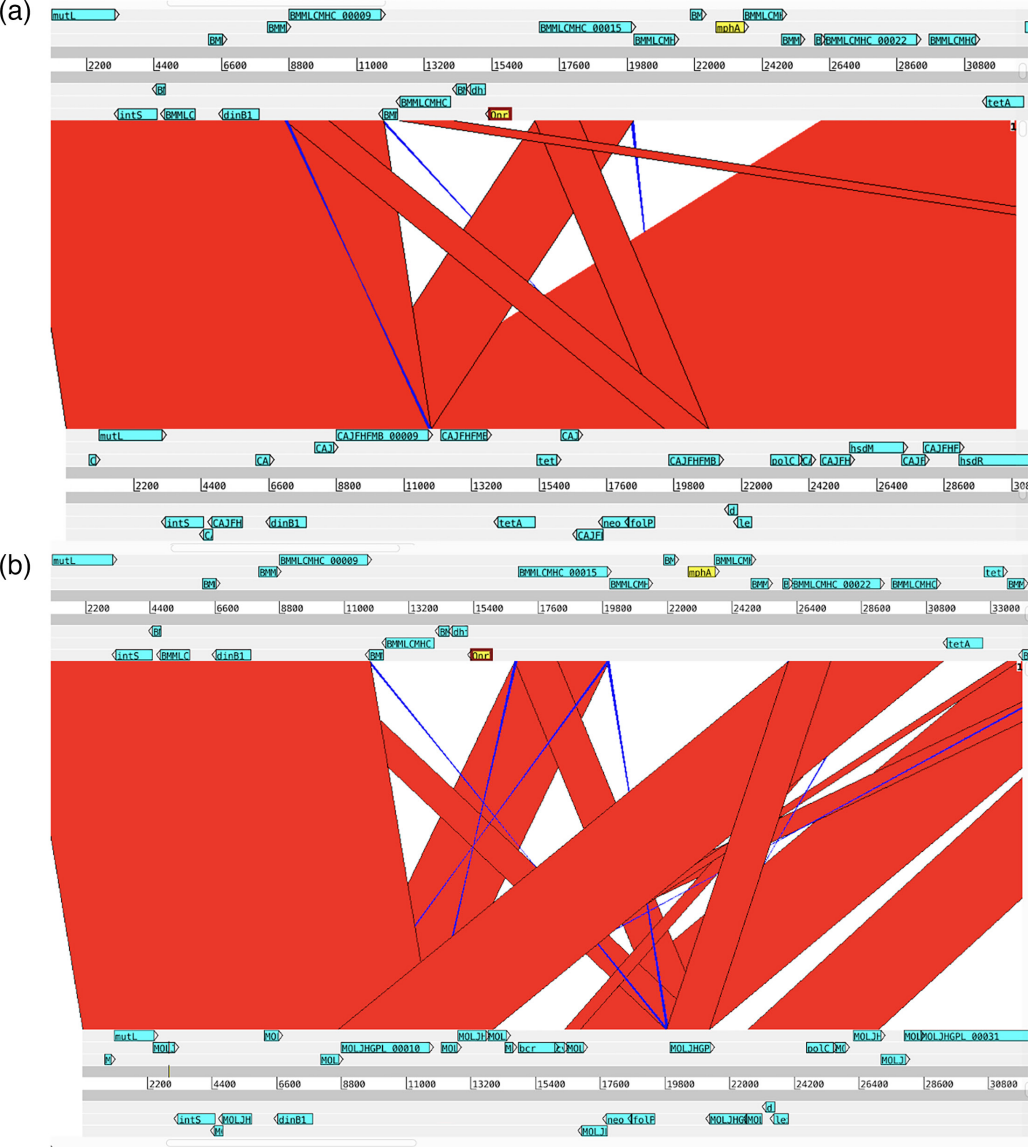
Local genome comparison plot of the ICE region containing AMR genes lost between 2006 and 2018. (**a**) *Vch*Ind6 from a 2006 BD-2 isolate (EN-1443) that contains *qnr_Vc_* and *mphA* compared to *Vch*Ind6 from a 2018 BD-2 isolate (EN-1581). (**b**) *Vch*Ind6 from EN-1443 compared to *Vch*Ind5 from another 2018 BD-2 isolate (EN-1469). Red blocks show aligned regions in the same order, whereas blue blocks show reversed aligned regions. Two important AMR genes absent from the 2018 genomes are coloured yellow.

Notably, two AMR genes on the SXT-R391 ICE (*qnr_Vc_* and *mphA*) that were previously associated with ciprofloxacin and azithromycin resistance in a 2006 sampling of *V. cholerae* genomes from Dhaka were absent in the 2018–2019 genomes [17]. To compare the structure of the SXT-R391 ICEs that harboured these genes, their sequences were obtained by extracting bases between the genes *prfC* and *gmr*, flanking the ICE insertion site. A *Vch*Ind6 from a 2006 BD-2 isolate (EN-1443) that contains *qnr_Vc_* and *mphA* was compared to another *Vch*Ind6 (EN-1581) from a 2018 BD-2 isolate (Fig. 5a). All *Vch*Ind6 from 2018 share this loss of *qnr_Vc_* and *mphA*. A 2018 *Vch*Ind5 ICE from a 2018 genome also contained this deletion relative to the ICE from 2006 (Fig. 5b). Thus, *qnr_Vc_* and *mphA* were absent in the 2018–2019 genomes due to one or more deletion events of an ICE region that was present in 2006 [17]. It is unclear if these genes were deleted due to fitness costs or if they were substituted for more effective resistance mechanisms elsewhere in the genome. In terms of exposures, ciprofloxacin was detected by mass spectrometry in 48 out of 51 stool samples (94%) from patients in a 2006 icddr,b cohort, but azithromycin was not detected [17]. In our 2018–2019 stool samples, ciprofloxacin detection was generally low, rarely exceeding 40% in a given month (Fig. S5), and was detected in 70 out of 273 samples overall (25.6%) and 12 out of 107 at the icddr,b (11.2%). While this suggests that inconsistent exposure to ciprofloxacin could potentially explain the loss of resistance genes, it is unclear if the detection rates in 2006 and 2018 are comparable or representative. Moreover, azithromycin detection rates were higher in 2018 (Fig. S5) compared to the lack of detection in 2006, suggesting that azithromycin could impose stronger selection for resistance. These exploratory hypotheses need further investigation in future targeted prospective studies.

### Variation in phage-defence gene profiles

We identified known phage-defence genes in our genomes using DefenseFinder [43]. Despite being relatively distantly related (Fig. 1), BD-1 and BD-3 genomes all contain 13 or 14 of 15 known defence systems (Fig. 4b). This suggests that defence systems such as *Abi*, BREX and Lamassu were present in the common ancestor of BD-1, 2 and 3 and were lost in BD-2. Other phage-defence systems such as DdmDE, Zorya and restriction–modification systems are universal to all sequenced genomes (Fig. 4b). The Lamassu system, commonly referred to as DdmABC in the *Vibrio* literature, acts in conjunction with *Abi* to kill phage-infected bacterial cells before phage replication can be completed, thereby providing population-level immunity [11]. BD-1 and BD-2 encode different variants of VSP-II, where DdmABC is encoded, likely explaining the lineage-specific differences [47]. Consistent with a prior study linking the BREX defence system to *Vch*Ind5, BREX genes were present in all *Vch*Ind5-containing genomes (BD-1 and BD-3) but none of the BD-2 isolates [14] (Fig. 4b). Together, these results show that co-circulating lineages have distinct profiles of both antibiotic and phage resistance genes which may offer diverse means of adapting to varied antimicrobial exposures.

## Discussion

In this study, we analysed *V. cholerae* genomes from cholera patients across Bangladesh over a 1-year outbreak season to assess genetic diversity using long-read sequencing. This revealed substantial diversity of different *V. cholerae* lineages encoding distinct profiles of AMR and phage-resistance genes co-circulating across the country. Although the samples collected covered a wide geographical region across Bangladesh, the sample size per month was still rather small, and from early 2019 onwards, isolates were exclusively sourced from icddr,b hospital in Dhaka. Despite these limitations, we identified an intriguing north–south gradient of genomic diversity, with a dominant lineage likely introduced from India from the north and greater diversity persisting in the south. This pattern merits further investigation within larger and longer sampling efforts. Differences in the local biotic or abiotic environment, human population density, migration rates, or cultural practices may explain some of the patterns observed.

We demonstrated the utility of long-read nanopore sequencing to resolve structural variation in *V. cholerae* genomes and to infer phylogenetic relationships. The number of SNVs in the long-read genomes was comparable to those in short-read genomes over similar spatial and temporal scales, suggesting that sequencing errors are not excessive in the long-reads after quality filtering [7]. The benefit of long-read sequencing technology is that large-scale genomic structures can be resolved at higher accuracy – for instance, we observed the deletion of SXT-ICE in a sub-lineage. Of the 273 genomes sequenced, 65% were assembled into two circular contigs, corresponding to the two chromosomes in *V. cholerae*, with the remaining assemblies failing to yield closed chromosomes. With DNA extraction protocols optimized for long reads, the completeness of assemblies could be further improved in future analyses. In contrast with the prevalent chromosome fusion observed in a 2015–2018 sample collection of *V. cholerae* long-read genomic sequences from Dhaka, we did not find evidence for fusion in our 2018–2019 sampling [44]. This could be due to fusion being possible in BD-2, but not BD-1, which lacks the homologous sequences on both chromosomes required for fusion. Our 2018–2019 sampling was dominated by BD-1, perhaps limiting our ability to identify fusion events. The factors selecting for fusion, therefore, remain unclear and merit further investigation.

Two distinct *V. cholerae* lineages, BD-1 and BD-2, have been predominant in Bangladesh since the late 1990s [9]. Between 1999 and 2017, BD-2 increased in frequency compared to BD-1 [9], until the BD-1.2 sub-lineage emerged as the cause of a major outbreak in 2022. Filling a gap between 2017 and 2022, our analysis shows that BD-1.2 dominated BD.2 throughout 2018, particularly in the north of Bangladesh. However, BD.2 persisted through the end of our sampling period in 2019. Our phylodynamic and geographic analyses support an Indian origin of BD-1.2, which likely arrived in Bangladesh from the north. Furthermore, we report a new lineage, BD-3, found only at the icddr,b sampling site in Dhaka. This lineage appeared throughout the sampling period but became particularly frequent in 2019, when it comprised the majority of genomes at icddr,b. BD-3 has a similar AMR and phage-resistance gene profile as BD-1, suggesting that it could compete with BD-1 in the future. Ongoing monitoring will be required to determine if this occurs.

Monir *et al.* suggested serotype switching as a possible explanation for the success of BD-1.2 over BD-2 [10]. Although we observed higher proportions of *wbeT* genotype associated with Ogawa serotype towards the end of our sampling period in early 2019, some isolates with Inaba-related *wbeT* persisted. The O-antigen may be under negative-frequency-dependent selection, such that no serotype reaches fixation and diversity is maintained [50]. Indeed, Ogawa and Inaba serotypes have co-existed in O1 7PET since the beginning of the seventh pandemic in the 1960s, and serotype switching in both directions has been frequently observed [36,37, 51].

Negative frequency-dependent selection could also arise from the co-evolution of *V. cholerae* and its virulent phages. *V. cholerae* lineages differ in the types of ICE they encode, and some genomes lack an ICE entirely [14]. Different ICE types encode different genes involved in both phage and antibiotic resistance [14]. For example, both BD-3 and BD-1 contained *Vch*Ind5, whereas BD-2 was split into sub-lineages that either possessed *Vch*Ind6 or lacked an ICE. A significant proportion of the isolates lacked an SXT-ICE despite the potential benefits it confers, such as antibiotic and phage resistance [14,18]. LeGault *et al.* posited that it may be beneficial for a *V. cholerae* population to include multiple lineages with distinct ICEs to increase the diversity of defence mechanisms against the ICP1 phage [14]. Strains without ICEs may be beneficial at the population level, as ICP1 released from ICE-free *V. cholerae* lacks epigenetic modifications necessary to evade ICE-encoded phage-restriction systems, thereby reducing the overall burden of phage predation [13,14]. Our data are consistent with the hypothesis that negative frequency-dependent selection favours rare ICE types, maintaining diversity in the population. This hypothesis is broadly supported by a recent study, reported shortly after our own, describing rapid gain and loss of mobile elements – particularly those involved in phage defence – in *V. cholerae* within Bangladesh [52]. Notably, many of these elements tended to be lost upon transmission outside Bangladesh, suggesting that their benefits may be restricted to regions of putatively high phage pressure. By contrast, another recent study proposed that anti-phage systems likely contributed to the successful transmission of a *V. cholerae* lineage from West Africa to South America, arguing that phage resistance may not always impose a fitness cost, preventing global transmission [53]. Understanding the role of *V. cholerae*-phage coevolution in explaining its local and global spread, therefore, remains a key challenge.

Antibiotic resistance has become widespread in clinical *V. cholerae*. A study of 443 *V*. *cholerae* isolates in the endemic region of India found that more than 99% of these were multidrug-resistant [54]. Consistent with this observation, 16 known AMR genes were identified in our data set, likely conferring resistance to a wide variety of antibiotics – including aminoglycoside, sulphonamide, trimethoprim, vancomycin, carbapenem, fluoroquinolone, macrolide, colistin, chloramphenicol and tetracycline. Each genome contained at least 10 of these 16 genes. The acquisition of AMR in *V. cholerae* has likely occurred via mobile elements such as ICEs, which can disseminate AMR genes between genetically distant strains [54]. In our data set, distinct ICE variants are associated with distinct lineages in a stable fashion, with occasional ICE gain or loss within a lineage. A variable ICE region containing two genes strongly associated with ciprofloxacin and azithromycin resistance in 2006, *qnr_Vc_* and *mphA*, was completely absent in the 2018–2019 genomes. Extensive gene conversions have been observed between SXT-ICEs, which could provide a mechanism for these deletions [18]. The absence of canonical ciprofloxacin and azithromycin resistance genes suggests that *V. cholerae* strains may rely on alternative mechanisms to maintain resistance to both drugs. Supporting the existence of other cryptic resistance mechanisms, the minimal inhibitory concentration of *V. cholerae* to both drugs has been steadily increasing since 2006, leading to reduced susceptibility [55,56]. Further study will be needed to determine if contemporary *V. cholerae* remain resistant to these drugs, and if so, by which genetic mechanisms.

In summary, our results highlight the genetic diversity present in clinical *V. cholerae* across Bangladesh over a 1-year outbreak season. In contrast to the lineage replacement events that appear to take place over decades in subsequent waves of globally successful clones, we observed that multiple distinct lineages, with distinct gene content, coexist within a year on a nationwide scale [3]. The north–south gradient of genomic diversity suggests geographically structured environmental pressures and transmission routes, which deserve further study. The scale of *V. cholerae* diversity within this single 1-year outbreak likely reflects the interplay between lineage transmission, competition and adaptation to selective pressures, including phages and antibiotics, which vary across time and space. Dissecting the nature and interactions among these selective pressures is a challenge for future research.

## Supplementary material

10.1099/mgen.0.001437 Uncited Supplementary Material 1.

10.1099/mgen.0.001437 Uncited Table S1.

10.1099/mgen.0.001437 Uncited Table S2.

10.1099/mgen.0.001437 Uncited Table S3.

## Abbreviations

AMR: antimicrobial resistance
CARD: Comprehensive Antibiotic Resistance Database
GC: genomic cluster
ICEs: integrative and conjugative elements
LB: Luria–Bertani
mHDM: mHealth Diarrhoea Management
NCS: National Cholera Surveillance
7PET: seventh Pandemic El Tor
SNVs: single-nucleotide variants
