# Supplementary material for: Vibrio cholerae lineage and pangenome diversity vary geographically across Bangladesh over 1 year
Source: Microb Genom. 2025 Jul 25;11(7):001437. doi: 10.1099/mgen.0.001437 (PMC12452190; doi:10.1099/mgen.0.001437)
Supplement: Uncited Supplementary Material 1. [file mgen-11-01437-s001.pdf]

Supplementary materials

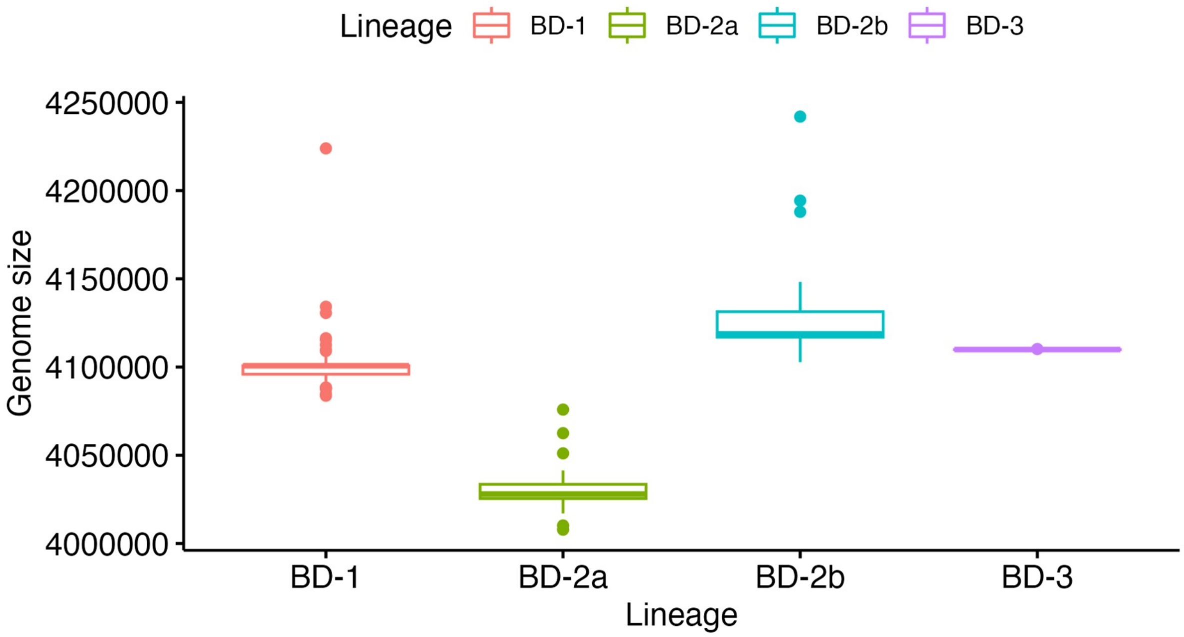

Supplementary Fig. 1: Distribution of genome assembly length by *V. cholerae* lineage. Boxplots showing the genome size distribution on the *Y*-axis which are grouped by lineages along the *Y*-axis. Lineages are also indicated by different colours.

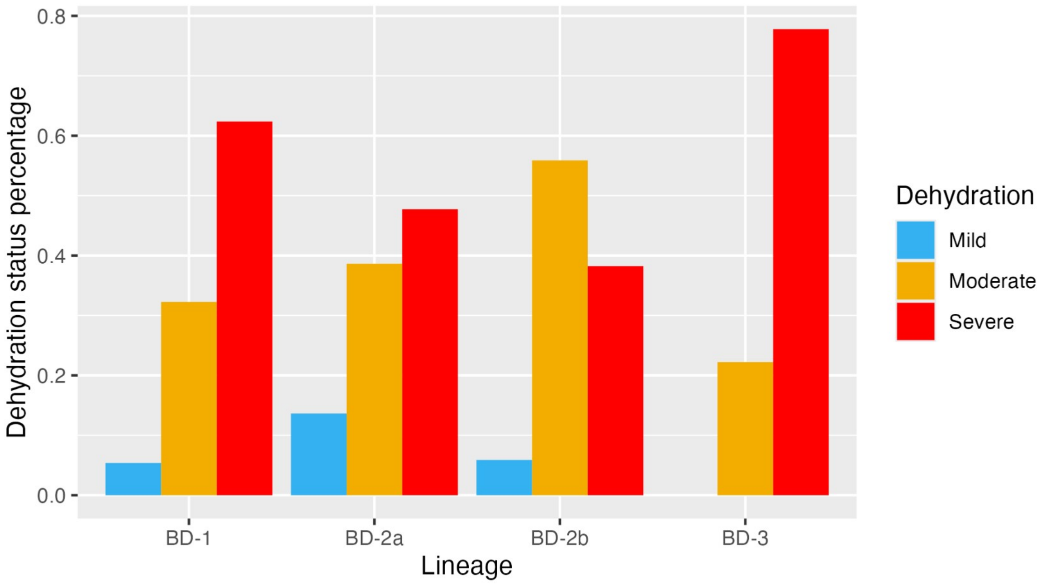

Supplementary Fig. 2: Distribution of dehydration severity across *V. cholerae* lineages. Bar plot showing the percentage of patient dehydration status within each lineage. The dehydration status was categorised into three levels: mild, moderate, and severe.

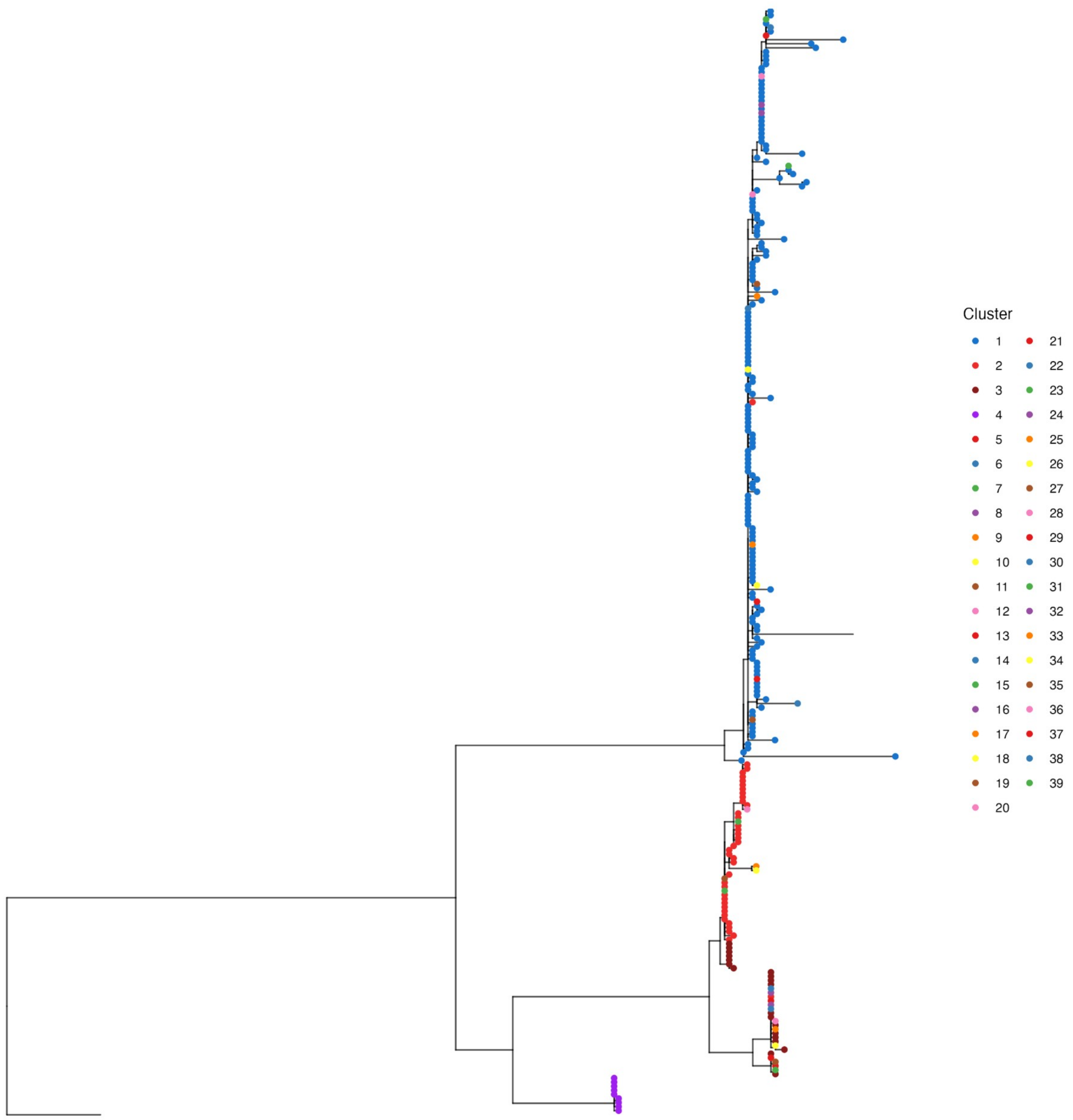

Supplementary Fig. 3: PopPUNK clusters mapped onto the phylogeny of newly-sequenced *V. cholerae* genomes. The maximum likelihood tree was rooted with a 1971 Bangladesh 7PET isolate as the outgroup (ERR025385). Leaves are coloured according to PopPUNK clusters, Clusters 1 to 4 were coloured using the same scale as the corresponding BD lineages in **Fig. 1**. Clusters 5 to 39 are singletons that mostly cluster within BD-1, BD-2a or BD-2b, but contain distinct accessory genome contents and are shown in different colours.

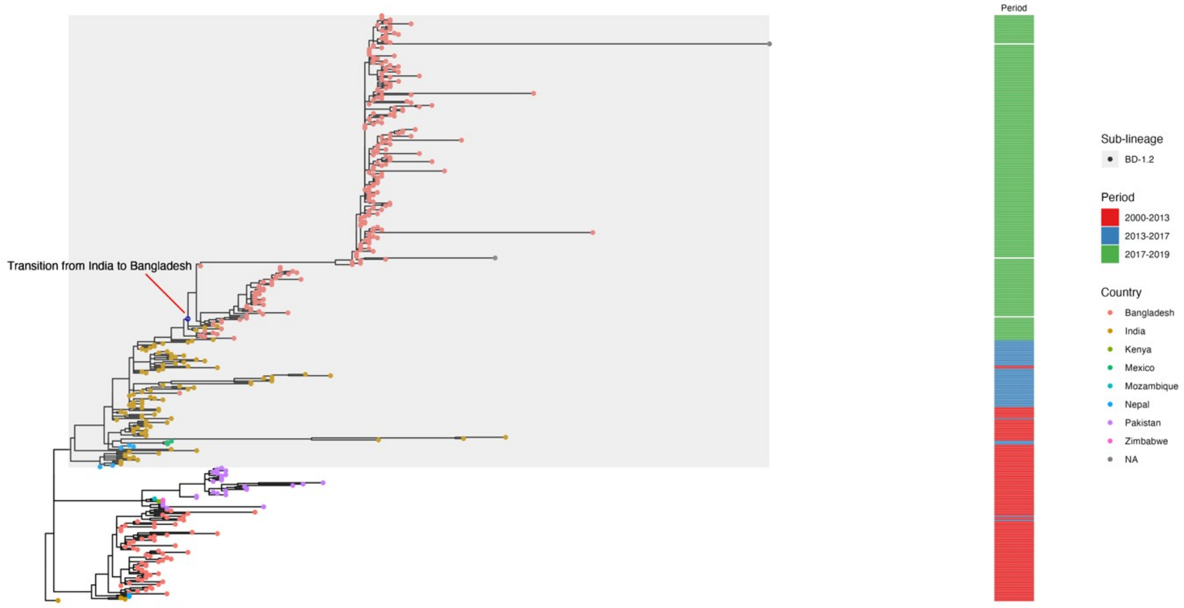

Supplementary Fig. 4: Phylogeny of BD-1 sampled from 2000 to 2019. The phylogeny is a subtree of **Fig. 1A** containing all BD-1.2 genomes from this study along with publicly available BD-1.1 and BD-1.2 genomes sampled from various different countries (shown as coloured points at the tree tips). The BD-1.2 sub-lineage is shaded in a grey box. The heatmap shows the time period in which each isolate was collected. The red arrow points to the common ancestor of the branch containing all BD-1.2 isolates from this study, along with other BD-1.2 in publicly available genome sequences. A maximum-likelihood estimation using PastML inferred its Indian origin (P = 99.67%).

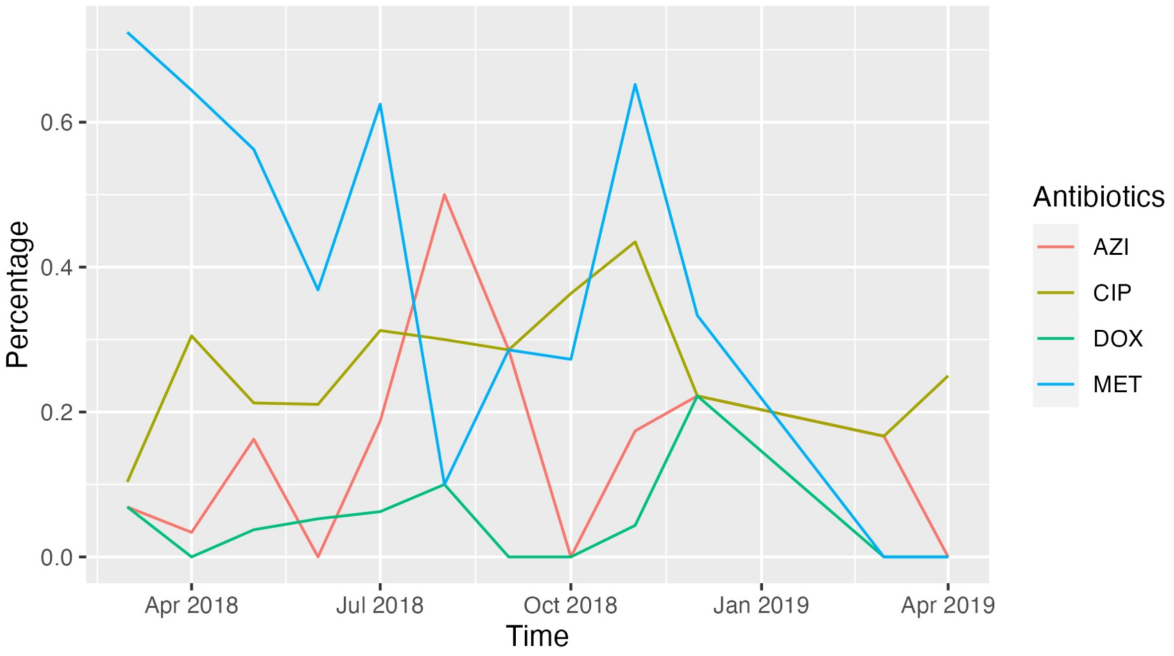

Supplementary Fig. 5: LCMS detection rate of four antibiotics over the sampling period. Line plots showing the percentage of samples with each of the antibiotics detected: AZI, azithromycin; CIP, ciprofloxacin, DOX, doxycycline, and MET, metronidazole. Detection counts are summed over all sampling locations across Bangladesh.

Supplementary Table 1: Metadata of newly sequenced and publicly available genomes used to construct the phylogeny in **Fig. 1**. Columns contain information on the year of isolation, the country of origin, the data set, and the lineage.

Supplementary Table 2: Comparison of serotypes predicted by antibody assay and *wbeT* genotypes. The first two columns contain the sample ID and the lineage. The third column shows the antibody assay predicted serotype, whereas the fourth column shows whether the *wbeT* gene is intact or disrupted by ISSpu7.

Supplementary Table 3: The first sheet shows the presence of AMR hits from the CARD-RGI output, '1' denotes presence and '0' denotes absence. The second sheet shows the annotation for each AMR hit, including information on frequency in the data set, the drug class and antibiotics affected, and the resistance mechanism.
